# Gla-Rich Protein Is a Potential New Vitamin K Target in Cancer: Evidences for a Direct GRP-Mineral Interaction

**DOI:** 10.1155/2014/340216

**Published:** 2014-05-18

**Authors:** Carla S. B. Viegas, Marjolein Herfs, Marta S. Rafael, José L. Enriquez, Alexandra Teixeira, Inês M. Luís, Cynthia M. R. van ‘t Hoofd, Alexandre João, Vera L. Maria, Sofia Cavaco, Ana Ferreira, Manuel Serra, Elke Theuwissen, Cees Vermeer, Dina C. Simes

**Affiliations:** ^1^Centre of Marine Sciences (CCMAR), University of Algarve, Campus de Gambelas, 8005-139 Faro, Portugal; ^2^GenoGla Diagnostics, Centre of Marine Sciences (CCMAR), University of Algarve, 8005-139 Faro, Portugal; ^3^VitaK, Maastricht University, 6229 EV Maastricht, The Netherlands; ^4^Algarve Medical Centre, Department of Histopathology, 8000-386 Faro, Portugal; ^5^Lisbon Central Hospital-CHLC, Department of Dermatology, 1169-050 Lisbon, Portugal; ^6^Administração Regional de Saúde do Algarve, 8135-014 Faro, Portugal; ^7^Private Hospitals of Portugal, HPP-Santa Maria Hospital, 8000-140 Faro, Portugal

## Abstract

Gla-rich protein (GRP) was described in sturgeon as a new vitamin-K-dependent protein (VKDP) with a high density of Gla residues and associated with ectopic calcifications in humans. Although VKDPs function has been related with ***γ***-carboxylation, the Gla status of GRP in humans is still unknown. Here, we investigated the expression of recently identified GRP spliced transcripts, the ***γ***-carboxylation status, and its association with ectopic calcifications, in skin basal cell and breast carcinomas. GRP-F1 was identified as the predominant splice variant expressed in healthy and cancer tissues. Patterns of ***γ***-carboxylated GRP (cGRP)/undercarboxylated GRP (ucGRP) accumulation in healthy and cancer tissues were determined by immunohistochemistry, using newly developed conformation-specific antibodies. Both GRP protein forms were found colocalized in healthy tissues, while ucGRP was the predominant form associated with tumor cells. Both cGRP and ucGRP found at sites of microcalcifications were shown to have *in vitro* calcium mineral-binding capacity. The decreased levels of cGRP and predominance of ucGRP in tumor cells suggest that GRP may represent a new target for the anticancer potential of vitamin K. Also, the direct interaction of cGRP and ucGRP with BCP crystals provides a possible mechanism explaining GRP association with pathological mineralization.

## 1. Introduction

Gla-rich protein (GRP), also known as cartilage matrix associated protein or upper zone of growth plate and cartilage matrix associated protein (UCMA) [1–4], was identified in sturgeon as a new vitamin-K-dependent protein (VKDP), exhibiting an unprecedented high density of Gla residues (16 Gla residues among 74 amino acids) and a high affinity for calcium mineral [1]. Highly conserved GRP orthologs presented conserved features specific to all VKDPs, in particular a remarkably well conserved Gla domain, thus suggesting GRP to be a new vertebrate-specific *γ*-carboxylated protein [1]. While in sturgeon GRP was predominantly found in cartilaginous tissues [1], in mammals it was shown to have a wider tissue distribution and to accumulate both in skeletal and connective tissues including bone, cartilage, skin, and vasculature [1, 2]. GRP was found to be a circulating protein and to be associated with calcifying pathologies affecting skin and arteries, where it accumulates at sites of ectopic calcifications and colocalizes with calcium mineral deposits [2]. Although the function of GRP is still unknown, it has been suggested to act as a negative regulator of osteogenic differentiation [4], a modulator of calcium availability in the extracellular matrix [1, 2], and as a potential inhibitor of soft tissue calcification in connective tissues [2]. In concordance, recent functional studies pointed to an essential role of GRP in zebrafish skeletal development and calcification [5], albeit GRP-deficient mice did not reveal evident phenotypic alterations in skeletal architecture, development, or calcification [6]. While four alternatively spliced transcripts of the GRP gene (GRP-F1, -F2, -F3, and F4) were described in mouse chondrocytes [7] and zebrafish [5, 8], new alternatively spliced transcripts were recently identified in humans. Besides GRP-F1, the new variants GRP-F5 and GRP-F6 were characterized by the loss of full *γ*-carboxylation and partial secretion functional motifs, due to deletion of exon 3 in F5 and exons 2 and 3 in F6 [9].

Although the precise function of the novel human GRP variants and the importance of their *γ*-carboxylation need to be further addressed, the perception that crucial differences exist between mouse and human GRP highlights the need for additional characterization of GRP expression/accumulation patterns in health and disease. Considering VKDPs for which the function is known, *γ*-carboxylation was shown to be required for biological activity [10–12], and under- or uncarboxylated species are generally regarded as proteins with low or no functional activity [13, 14]. Several factors, such as insufficient dietary intake of vitamin K, mutations in the *γ*-glutamyl carboxylase enzyme (GGCX), and warfarin treatment, result in decreased *γ*-carboxylation of VKDPs, which has been associated with an increased risk for osteoporotic bone loss [15, 16] as well as for arterial and skin calcifications [12, 17–20]. Microcalcifications are also often associated with different types of cancer and are considered as a hallmark for early detection of breast cancer, with recognized prognostic relevance. Accumulating evidence suggests that mineralization occurring in breast cancer is a cell-specific regulated process sharing molecular mechanisms involved in arterial pathological mineralization and physiological mineralization in bone [21]. Several reports have described an association of VKDPs with different types of cancer, namely, matrix Gla protein (MGP) and uncarboxylated prothrombin (des-*γ*-carboxy prothrombin, DCP) [22–28]. Although a relation between MGP *γ*-carboxylation status and neoplasias is still unknown, for prothrombin it was shown that increased circulating DCP can be used as a diagnostic and prognostic marker for hepatocellular carcinoma [22, 28, 29]. It is remarkable that, despite the widely reported vitamin K anticancer potential [[10, 30], and references therein], its mechanism of action in cancerous processes remains elusive. Therefore, the identification of new vitamin K targets in cancer, such as GRP, may contribute to unveil the role and functional mechanism of vitamin K in cancer development.

Here, we report on the association of GRP in human skin and breast cancers with the specific accumulation pattern of carboxylated (cGRP) and undercarboxylated (ucGRP) protein forms. In these studies we used newly developed conformation-specific antibodies against ucGRP and cGRP. We investigated the association and *γ*-carboxylation status of GRP with microcalcifications occurring in skin and breast cancers and tested the mineral binding capacity of both protein forms, which may help understanding the mechanism behind the previously reported association of GRP with pathological mineralization.

## 2. Materials and Methods

### 2.1. Ethics Statement

This study complies with the guidelines for good clinical practice and was performed in accordance with the Declaration of Helsinki and approved by the ethics committees of all the hospitals and institutions involved, namely Algarve Medical Centre, Lisbon Central Hospital, HPP-Santa Maria Hospital, and National Institute of Legal Medicine and Forensic Sciences, Public Institute. Written informed consent was obtained from all participants.

### 2.2. Biological Material

Control breast (mammary gland, MG) and skin (Sk) tissues were obtained from three healthy volunteers at the time of esthetic surgeries. Nontumorous areas, adjacent to tumors, present in four skin and four breast cancer patient samples, were also used as controls in immunohistochemical (IHC) staining experiments. Samples of malignant breast lesions were retrieved from patients who had undergone breast surgery at the Algarve Medical Centre. Skin biopsies of malignant lesions were taken under local anesthesia at Lisbon Central Hospital. A total of eleven tissue samples from patients diagnosed with basal cell carcinoma (BCC, Table 1) and eleven from patients diagnosed with invasive ductal carcinoma (IDC, Table 2) were studied. IHC was performed in eight BCC and seven IDC samples, and gene expression analysis was performed in five BCC and four IDC samples.

### 2.3. Sample Processing

Tissue samples were embedded in paraffin at the Pathology Departments of Algarve Medical Centre and Lisbon Central Hospital and histologically classified by pathologists. Physiological structures were identified by regular haematoxylin-eosin staining and mineral deposits were detected with silver nitrate (Sigma-Aldrich) by the von Kossa method. Samples used in gene expression studies were collected into RNAlater (Sigma-Aldrich) immediately after surgery.

### 2.4. RNA Extraction

Total RNA was extracted from Sk, MG, BCC, and IDC tissues as described by Chomczynski and Sacchi [31]. RNA integrity was evaluated by agarose-formaldehyde gel electrophoresis and concentration determined by spectrophotometric analysis at 260 nm.

### 2.5. Gene Expression

One microgram of total RNA was treated with RQ1 RNase-free DNase (Promega) and reverse-transcribed at 37°C with MMLV-RT (Invitrogen) using a dT adapter. PCR amplifications for GRP-F1, -F5, and -F6 splice variants were performed with SsoFast EvaGreen Supermix (BioRad) for 50 cycles and specific primer sets A/B, C/D, and C/E, respectively. Ribosomal 18S was used as loading control. A list of all PCR primer sequences is presented in Table 3.

### 2.6. Quantitative Real-Time PCR (qPCR)

Quantitative PCR was performed with an iCycler iQ apparatus (Bio-Rad) using 25 ng cDNA and the conditions described above. In addition to GRP-F1, -F5, -F6 and 18S, MGP, GGCX, VKOR (vitamin K epoxide reductase), OPN (osteopontin), TNF*α* (tumor necrosis factor alpha), and GAPDH were amplified using primer sets as described in Table 3. Fluorescence was measured at the end of each extension cycle in the FAM-490 channel and melting profiles of each reaction were performed to check for unspecific product amplification. Levels of gene expression were calculated using the comparative method (ddCt) and normalized using gene expression levels of both GAPDH and 18S housekeeping genes, with the iQ5 software (BioRad); qPCR was performed in duplicates and a normalized SD was calculated.

### 2.7. Conformation-Specific Antibodies against Carboxylated (cGRP) and Undercarboxylated (ucGRP) GRP Protein Forms

**Figure 1:**
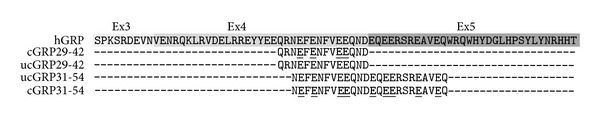
Conformation-specific antibodies for *γ*-carboxylated (cGRP) and undercarboxylated (ucGRP) GRP forms. Amino acid sequence alignments of mature hGRP-F1 (hGRP) and synthetic peptides used as antigens for development of conformational c/ucGRP antibodies: cGRP29-42 and ucGRP31-54, respectively, and ucGRP29-42 and cGRP31-54 peptides used to test respective conformational specificity. GRP exons (Ex3, Ex4, and Ex5) are denoted in the corresponding amino acid sequence in gray scale; bold and underlined E in cGRP29-42 and cGRP31-54 indicate Gla residues.

Affinity-purified chicken polyclonal antibody against cGRP (cGRP pAb) (GenoGla Diagnostics, Faro, Portugal) was produced by immunizing chickens with a synthetic peptide corresponding to a *γ*-carboxylated region of the human GRP Gla-domain located within exon 4 (aa 29-42: QRNEFENFVEEQND, in which all E are Gla-residues and termed cGRP29-42, Figure 1). An equivalent, but noncarboxylated peptide (aa 29-42, where all E are Glu residues), was termed ucGRP29-42 (Figure 1). The conformation-specific affinity-purified antibody was obtained by passing the chicken serum through an ucGRP29-42 affinity column followed by immunopurification of the flow-through on a cGRP29-42 column.

Monoclonal antibody against ucGRP (ucGRP mAb) (VitaK BV, Maastricht, The Netherlands) was raised against an epitope of human GRP located within exons 4 and 5 (aa 31-54: NEFENFVEEQNDEQEERSREAVEQ), in which all E are Glu residues and termed ucGRP31-54 (Figure 1). An equivalent, but carboxylated peptide (aa 31-54, where all E are Gla residues), was termed cGRP31-54 (Figure 1). The conformation-specific ucGRP mAb was raised in BALB/c mice and postimmune sera were screened for their conformational affinity toward synthetic c and ucGRP31-54 peptides. After a satisfying antibody titer was reached, splenocytes were fused with a mouse myeloma cell line (Sp 2/01-Ag, CRL 8006, ATCC). Clones strongly reacting with ucGRP31-54 and nonreactive with cGRP31-54 were selected, and the antibodies produced were purified by protein G affinity chromatography.

CTerm-GRP polyclonal antibody (GenoGla Diagnostics) detecting total GRP was produced against a synthetic peptide corresponding to the C-terminus of rat GRP, following a previously described procedure [2].

### 2.8. Immunohistochemistry

Immunohistochemical staining was performed on paraffin-embedded tissue sections as described elsewhere [2]. Briefly, endogenous peroxidase activity was blocked with 3% (v/v) H_2_O_2_ in TBST buffer (TBST: 0.1 mol/L Tris, 0.15 mol/L NaCl, 0.1% (v/v) Triton X-100) for 15 min. Nonspecific antibody binding was blocked with TBT (0.5% (w/v) BSA in TBST) for 1 h at 37°C. Incubation with CTerm-GRP, cGRP pAb, and ucGRP mAb (5, 1, and, 1 *μ*g/mL diluted in TBT, resp.) was performed overnight (O/N) in a humidified chamber at room temperature (RT). Blocking assays were performed by incubating cGRP pAb (1 *μ*g/mL) with 10^-6 ^M of cGRP29-42 or ucGRP29-42 peptides and ucGRP mAb (1 *μ*g/mL) with 10^−6^ M of ucGRP31-54 or cGRP31-54 peptides, for 2 h at RT prior to tissue incubation. Primary antibodies were detected using species specific HRP-conjugated secondary antibodies (Sigma-Aldrich) and 0.025% (w/v) 3,3-diaminobenzidine (Sigma-Aldrich). Negative controls consisted in the substitution of primary antibody with TBT. Counterstaining was performed with haematoxylin. Microscopic images were acquired in a Zeiss AXIOIMAGER Z2 microscope, with an AxioCam ICc3 camera and AxioVision software version 4.8 (Carl Zeiss), at the light microscopy facility, Department of Biomedical Sciences and Medicine, University of Algarve (Portugal).

### 2.9. Cloning of hGRP-F1 into pET151 Expression Vector

The complete open reading frame (ORF) of the human GRP-F1 isoform (hGRP) was amplified by nested PCR from reverse transcribed Sk total RNA, using HsGRPCDS1_Fw and HsGRPCDS3_Rv specific primers in the first PCR, followed by nested amplification with HsGRPCDS2_Fw/HsGRPEx5R2 primers. PCR products were cloned into pCR^II^TOPO (Invitrogen) and sequenced (CCMAR sequencing facilities, Faro, Portugal). Human GRP cDNA coding for the secreted GRP-F1 protein was amplified using an Sk-derived positive clone and specific primers ReHsGRP_CFw/ReHsGRP_Rv designed to allow directional cloning into a pET151/D-TOPO vector (Champion pET Directional TOPO Expression kit, Invitrogen). A His_6_ tag, a V5 epitope, and a tobacco etch virus (TEV) protease cleavage site were added to the* N-terminus* of the expressed protein. Correct cloning was verified by DNA sequencing (CCMAR). A list of all PCR primer sequences is presented in Table 3.

### 2.10. Recombinant Protein Expression and Purification

*Escherichia coli* BL21star (DE3) cells (Champion pET Directional TOPO Expression kit) were transformed according to manufacturer's instructions and induction was performed with 1 mM IPTG for 4 h. Cells were pelleted by centrifugation, resuspended in binding buffer (20 mM sodium phosphate, 0.5 M NaCl, 20 mM imidazole, pH 7.4), and sonicated for 3 min in 10 sec pulses series at 60 V. The resulting cleared supernatant was loaded onto a 1 mL HisTrap HP column (GE Healthcare) according to manufacturer's instructions, and recombinant protein was eluted with 20 mM sodium phosphate, 0.5 M NaCl, 500 mM imidazole, pH 7.4. Recombinant human GRP (rhGRP) protein purity was assessed by SDS-PAGE.

### 2.11. Extraction and Purification of GRP and MGP from Calcified Tissues

Sturgeon GRP (sGRP) was extracted and purified as previously described [1]. Identification of purified protein, obtained after RP-HPLC purification, was confirmed by N-terminal amino acid sequence. Bovine MGP (bMGP) was extracted from bovine calcified costal cartilage, obtained from local slaughterhouse, as described [32]. Briefly, the formic acid demineralized fraction containing mineral-binding proteins was dialyzed against 50 mM HCl using 3,500 molecular weight tubing (Spectra/Por 3, Spectrum) over two days and then freeze-dried and dissolved in 6 M guanidine-HCl, 0.1 M Tris, pH 9.0. Subsequent partial purification was achieved by precipitation of insoluble proteins (mainly MGP) through dialysis against 5 mM ammonium bicarbonate. Precipitated MGP was dissolved in 6 M guanidine-HCl, 0.1 M Tris, pH 9.0. HisTrap rhGRP was further purified through RP-HPLC as described above for sGRP, and recombinant* Thermus thermophilus *S6 ribosomal protein (S6) was a kind gift from Professor Eduardo Melo (CBME, University of Algarve, Portugal).

### 2.12. Protein Mineral Complex (PMC)* In Vitro* Assay

Basic calcium phosphate (BCP) crystals were produced as previously described [33] by incubating 2 mM CaCl_2_ and 10 mM sodium phosphate buffer pH 7.0 for 2 h at 37°C and then centrifuged at 20 000 ×g for 20 min at RT. BCP crystals were incubated for 30 min at 37°C, with approximately 5 *μ*g of each protein (rhGRP, sGRP, bMGP, and S6) in 25 mM boric acid, pH 7.4. After centrifugation at 20 000 ×g for 20 min at RT, supernatants containing non-bound mineral proteins were collected, lyophilized, and analyzed by SDS-PAGE. Pellets containing PMCs were first washed with 25 mM boric acid, pH 7.4 and then demineralized with 30% (v/v) formic acid for 2 h at 4°C with agitation. After centrifugation at 20 000 ×g for 20 min at 4°C, the supernatant containing the BCP-binding proteins was collected, lyophilized, and analyzed by SDS-PAGE.

### 2.13. Electrophoresis and Dot-Blot Analysis

Aliquots of protein were separated on a 4 to 12% gradient SDS-PAGE (NuPage, Invitrogen) gel and proteins were visualized by Coomassie Brilliant Blue (CBB, USB) staining as described [2]. For dot-blot immunodetection, 100, 50, and 25 ng of synthetic peptides were applied onto a nitrocellulose membrane (BioRad), as described [2], and incubated O/N with a 1 : 1000 dilution of cGRP pAb and ucGRP mAb, respectively. Immunodetection was achieved using species-specific secondary horseradish-conjugated antibodies and Western Lightning Plus-ECL (PerkinElmer).

## 3. Results

### 3.1. GRP-F1 as Main GRP Splice Variant Expressed in Human Skin and Mammary Gland

**Figure 2:**
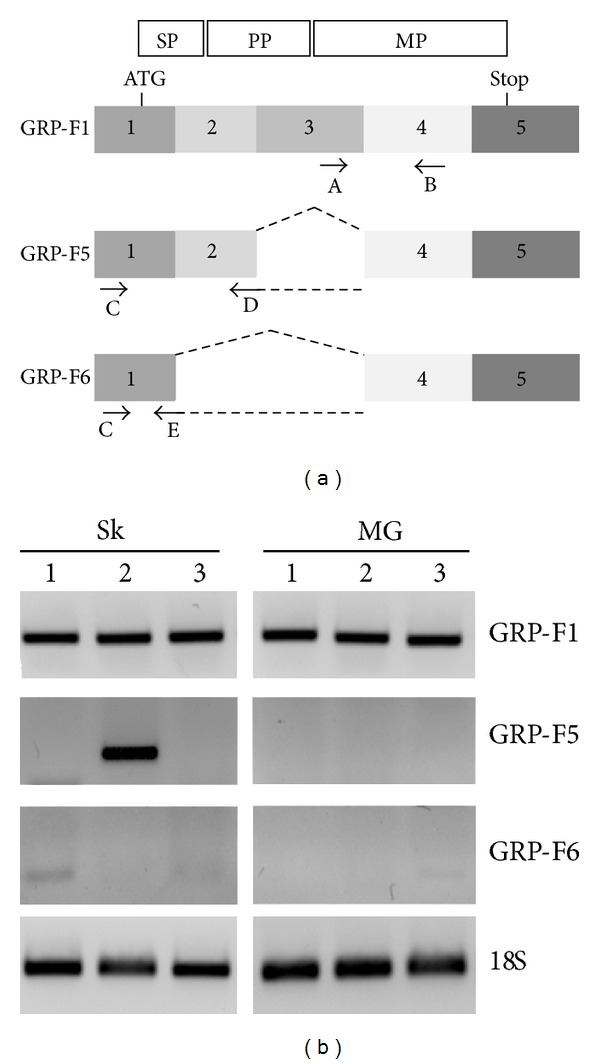
GRP-F1 is the predominant splice variant expressed in healthy human skin and mammary gland. (a) Schematic representation of GRP-F1, -F5, and -F6 splice transcripts and strategy to obtain corresponding transcript-specific primer sets. Structural organization of full length GRP-F1 is represented in white boxes above corresponding coding exons and limited to the open reading frame (between ATG and Stop codons): SP, signal peptide; PP, propeptide; MP, mature protein. (b) Gene expression analysis of GRP-F1, -F5, and -F6 splice transcripts in three control samples of both skin (Sk 1–3) and mammary gland (MG 1–3) tissues, using the above-described primer sets. 18S was used as loading control for sample integrity; transcript sizes are 118, 172, and 102 bp for F1, F5, and F6, respectively.

In order to study the expression pattern of GRP splice variants in skin (Sk) and mammary gland (MG) control tissues, specific primers were designed (Figure 2(a)) and tested for the unique amplification of each splice variant using GRP-F1, -F5, and -F6 clones. Primer sets A-B, C-D, and C-E were shown to specifically amplify GRP-F1, -F5, and -F6, respectively (results not shown). GRP-F1 was consistently amplified in all control samples of Sk and MG analyzed, while GRP-F5 and -F6 expressions were shown to be heterogeneous: barely detectable in some Sk samples and mostly undetectable in the MG tissues analyzed (Figure 2(b)). Overall, the expression pattern of GRP splice variants shows that the GRP-F1, coding for the full protein, is the main transcript present in control skin and mammary gland.

### 3.2. GRP Accumulates in Both Skin and Mammary Gland Control Tissues

**Figure 3:**
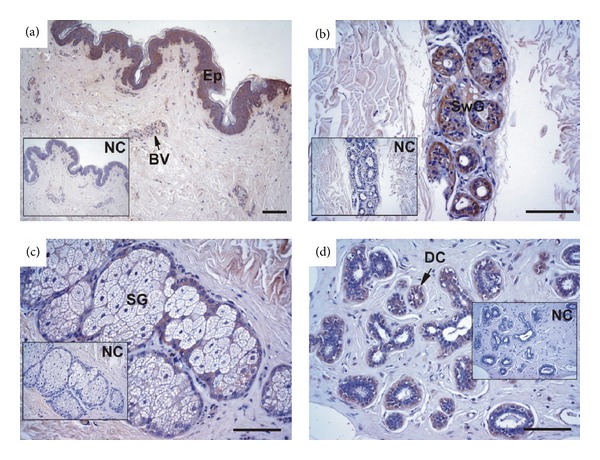
GRP accumulates in healthy skin and mammary gland tissues. Immunolocalization of total GRP was performed with the CTerm-GRP antibody in healthy skin (a–c) and mammary gland (d) tissue sections. In skin, GRP accumulates at the epidermis (Ep) and blood vessels (BV) (a), sweat (SwG, b) and sebaceous (SG, c) glands. In mammary gland, GRP accumulates in the cytoplasm of ductal cells (DC) forming the lobules (d). NC represents negative controls performed by omitting the CTerm-GRP antibody in consecutive tissue sections. Samples were counterstained with haematoxylin. Scale bar represents 100 *μ*m.

The pattern of total GRP accumulation was determined in control human skin (Figures 3(a)–3(c)) and mammary gland (Figure 3(d)). Strong positive staining for GRP was observed in all strata of the epidermis (Ep), in small blood vessels (BV) at the dermis level (Figure 3(a)), in hair follicles (results not shown) and in sweat (SwG; Figure 3(b)) and sebaceous (SG; Figure 3(c)) glands; this is consistent with the previously described pattern of GRP accumulation in human skin [2]. In normal mammary tissue, GRP was mainly detected in the cytoplasm of ductal cells (DC) forming the lobules (Figure 3(d)) and in small arterioles (results not shown). Negative controls (NC) showed absence of signal.

### 3.3. GRP-F1 and Genes Involved in *γ*-Carboxylation Share Gene Expression Pattern in Skin and Breast Cancers

**Figure 4:**
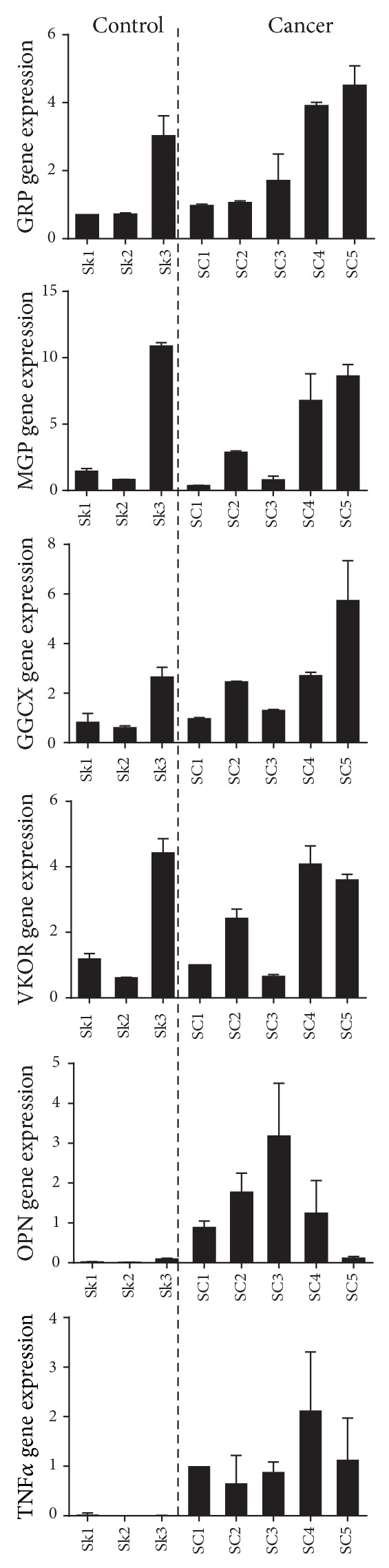
GRP-F1, MGP, and *γ*-carboxylation related genes exhibit similar expression patterns in control and skin cancer. Levels of GRP-F1, MGP, GGCX, VKOR, OPN, and TNF*α* gene expression were determined by qPCR in three control skin (Sk 1–3) and five skin cancer (SC 1–5) samples and normalized using 18S and GAPDH as housekeeping genes. Expression values are relative to zero and represent the mean of duplicates; standard deviations are indicated.

**Figure 5:**
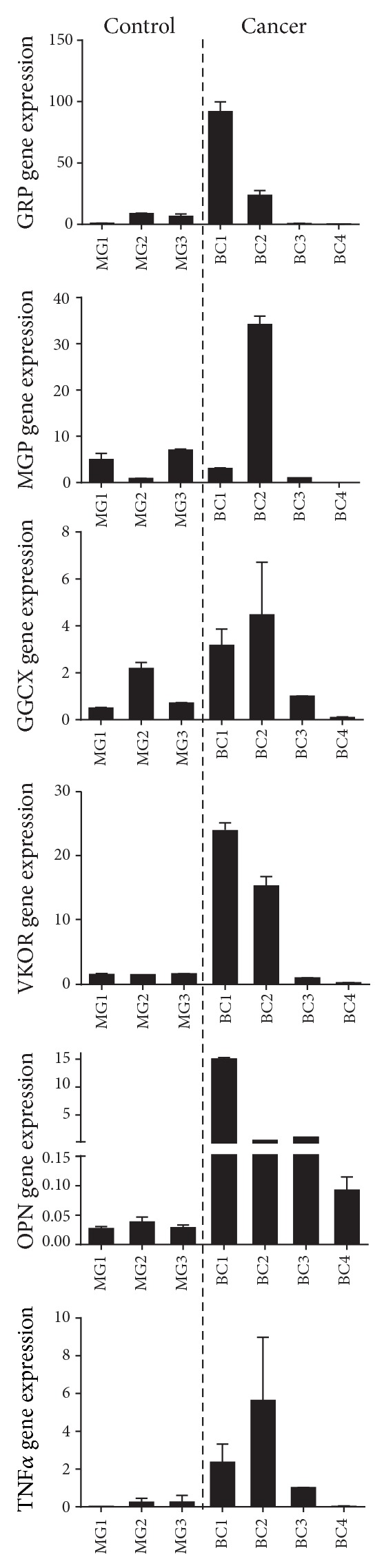
GRP-F1 is upregulated in mineralization-containing breast cancer samples. Levels of GRP-F1, MGP, GGCX, VKOR, OPN, and TNF*α* gene expression were determined by qPCR in three control mammary gland (MG 1–3) and four breast cancer (BC 1–4) samples and normalized using 18S and GAPDH as housekeeping genes. Expression values are relative to zero and represent the mean of duplicates; standard deviations are indicated.

Expression levels of GRP splice transcripts were determined in control and cancerous tissues and correlated with gene expression of MGP, GGCX, VKOR, and the tumor markers OPN and TNF*α* (Figures 4 and 5). Both in skin cancer (SC) and in the control samples (Sk), the levels of GRP-F1 were found to be heterogeneous without a clear tendency for up- or downregulation in cancer cases (Figure 4). Interestingly, the same heterogeneous pattern was found for MGP, GGCX, and VKOR, while OPN and TNF*α* were found clearly upregulated in tumor samples (Figure 4). These results suggest a concerted expression of the VKDPs, GRP and MGP, and the genes involved in the *γ*-carboxylation process, which cannot be associated with growth, progression, or metastasis of cancer processes at this time. Of notice, skin cancer samples analyzed were devoid of microcalcifications, as determined by von Kossa staining and confirmed by histological evaluation by pathologists (Table 1). Similar gene expression results were found in control MG and breast cancer (BC) samples (Figure 5), with heterogeneous levels of GRP-F1, MGP, GGCX, and VKOR and increased expression of OPN and TNF*α* in cancer cases (Figure 5). However, higher levels of GRP-F1, MGP, GGCX, and VKOR were found in BC samples that include microcalcifications (Table 1), suggesting an upregulation associated with calcification, but not necessarily with tumor development. Gene expression of GRP-F5 and -F6 transcripts was found to be nearly undetectable in the majority of samples from both skin and breast cancers (results not shown), highlighting the predominance of the GRP-F1 transcript in all tissues and conditions analyzed.

### 3.4. Validation of Novel Conformation-Specific Antibodies against Human Carboxylated (cGRP) and Undercarboxylated (ucGRP) GRP Protein Forms

**Figure 6:**
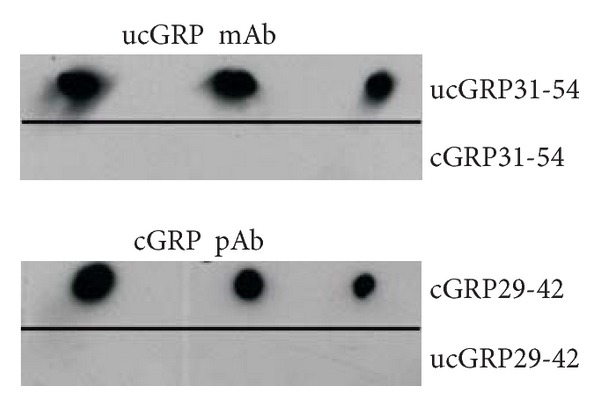
Validation of conformation-specific antibodies for cGRP and ucGRP forms. Conformation specificity of cGRP and ucGRP antibodies was tested by dot blot using synthetic peptides (100, 50, and 25 ng peptide each lane): cGRP29-42; ucGRP31-54; ucGRP29-42; and cGRP31-54, and shows specific detection of cGRP and ucGRP antibodies to cGRP29-42 and ucGRP31-54 peptides, respectively.

Conformational specificity of cGRP and ucGRP antibodies was initially screened by a cross-reactivity test of immune sera with each peptide in both *γ*-carboxylated and non-*γ*-carboxylated forms. Cross-reactivity of purified cGRP pAb and ucGRP mAb with all available synthetic peptides was further tested by dot-blot, confirming specificity of cGRP pAb for cGRPpep29-42 and of ucGRP mAb for ucGRPpep31-54 (Figure 6). In addition, quenching assays were performed for both antibodies to validate their use in IHC. Blocking recognition of cGRP pAb and ucGRP mAb epitopes was performed using the corresponding synthetic peptides in both forms; incubations of cGRP pAb with cGRPpep29-42 and ucGRP mAb with ucGRPpep31-54 resulted in a decreased signal intensity, while incubations of cGRP pAb with ucGRPpep29-42 and ucGRP mAb with cGRPpep31-54 showed similar signal staining as nonblocked antibody assays (results not shown).

### 3.5. Differential Accumulation Pattern of Human cGRP and ucGRP in Skin and Breast Cancers

**Figure 7:**
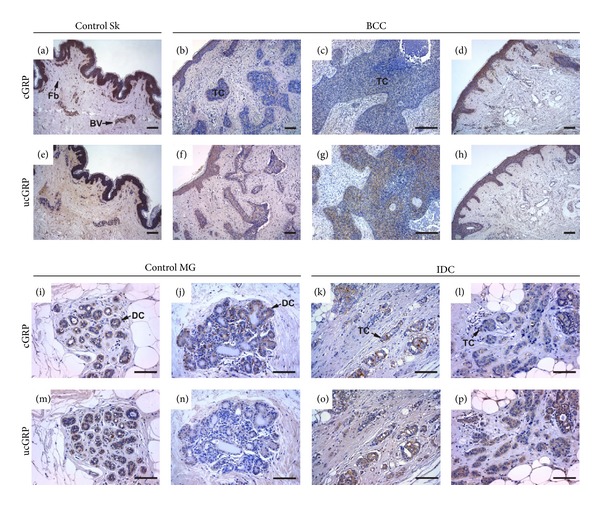
cGRP preferentially accumulates in healthy tissues while ucGRP is the predominantly form associated with tumor cells. Immunolocalization of cGRP and ucGRP in control skin (Sk; (a, e), resp.), basal cell carcinoma (BCC; (b–d), (f–h), resp.), control mammary gland (MG; (i–j), (m–n), resp.), and invasive ductal carcinoma (IDC; (k–l), (o–p), resp.) tissue sections was performed with cGRP and ucGRP antibodies, respectively. In control skin, cGRP and ucGRP are similarly accumulated in the epidermis, although cGRP is the predominant form in blood vessels (BV) and fibroblasts (Fb) ((a, e), resp.). In BCC tumor cells (TC), cGRP levels are significantly decreased (b, c), while ucGRP is the predominant form (f, g) compared to both healthy skin (a, e) and non-affected areas adjacent to tumor cells (d, h). In control mammary gland (MG), cGRP is accumulated in the ductal cells (DC; (i, j)), while ucGRP accumulation is either similar (m) or decreased (n) compared to cGRP. In IDC tumor cells, the amount of cGRP is significantly decreased (k, l) in relation to ucGRP (o, p). Sections were counterstained with haematoxylin. Scale bar represents 100 *μ*m.

Fifteen individual cases (eight skin and seven breast cancers, see Tables 1 and 2) were analyzed by IHC using cGRP and ucGRP antibodies and compared with control tissues (Figure 7). In control skin, cGRP and ucGRP (Figures 7(a) and 7(e), resp.) colocalized with total GRP (Figure 3(a)), although most of the fibroblasts (Fb) of the upper dermis were only stained for cGRP (Figure 7(a)). In BCC tumors, a clear difference is observed between cGRP (Figures 7(b) and 7(c)) and ucGRP (Figures 7(f) and 7(g)) accumulation associated with tumor cells (TC): staining for cGRP was decreased compared to ucGRP, while in healthy skin areas adjacent to tumors, both GRP forms are similarly accumulated (Figures 7(d) and 7(h)) with a pattern comparable to control skin (Figures 7(a) and 7(e)). These results indicate that although both GRP forms are present in control skin, cGRP is mainly present in the healthy tissue while ucGRP is the protein form predominantly occurring in association with tumor cells in BCC.

In healthy mammary glands cGRP was consistently detected in the cytoplasm of ductal cells (DC) forming the lobules (Figures 7(i) and 7(j)) and colocalized with total GRP (Figure 3(d)), while ucGRP was found to be either colocalized (Figure 7(m)) or undetectable (Figure 7(n)), depending on samples analyzed. In contrast, in IDC samples ucGRP was consistently detected throughout the cytoplasm of tumor cells (TC; Figures 7(o) and 7(p)), while cGRP was found to be highly localized with a pointed spot pattern to certain tumor cells (Figures 7(k) and 7(l)). Overall, ucGRP is the predominant form accumulating in IDC-tumor cells, while cGRP preferentially accumulates in healthy mammary gland. Of notice, not all areas of IDC analyzed were found positive for GRP, but in areas with a positive signal, the described patterns were always observed. Negative controls were performed for each antibody and each sample analyzed and showed absence of signal (results not shown).

### 3.6. Both cGRP and ucGRP Protein Forms Are Accumulated at Sites of Microcalcifications in BCC and IDC

**Figure 8:**
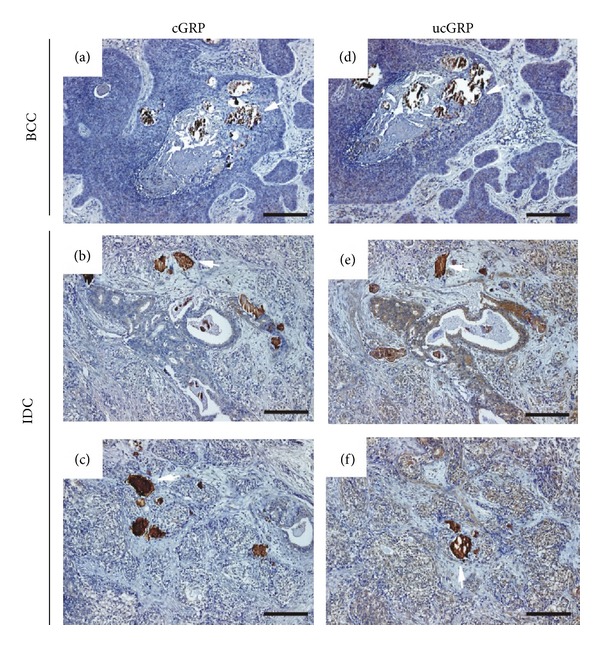
cGRP and ucGRP are highly accumulated at sites of microcalcifications in BCC and IDC. Sites of cGRP (a, b, c) and ucGRP (d, e, f) accumulation were determined by IHC in BCC (a, d) and IDC (b, c and e, f) tissue sections, using the c/ucGRP antibodies. Both GRP protein forms are highly accumulated at sites of mineral deposits in all BCC and IDC calcification-containing samples analyzed. White arrows show examples of microcalcifications; sections were counterstained with haematoxylin. Scale bar represents 100 *μ*m.

From all samples analyzed by von Kossa staining, two BCC and four IDC samples were found to contain microcalcifications (results not shown) and classified as light, moderate, or massive, according to the quantity and size of the mineral present (Tables 1 and 2). In all samples containing microcalcifications, both cGRP (Figures 8(a)–8(c)) and ucGRP (Figures 8(d)–8(f)) were detected colocalizing with mineral deposits in BCC (Figures 8(a) and 8(d)) and IDC (Figures 8(b), 8(c), 8(e), and 8(f)). These results strongly suggest that both cGRP and ucGRP have a high affinity for calcium mineral deposits.

### 3.7. *In Vitro* Association of Both c and ucGRP Protein Forms with Basic Calcium Phosphate (BCP) Crystals

Calcium/phosphate (Ca/P) mineral-binding capacity of carboxylated and noncarboxylated GRP protein forms was evaluated using protein mineral complex (PMC) assays.

**Figure 9:**
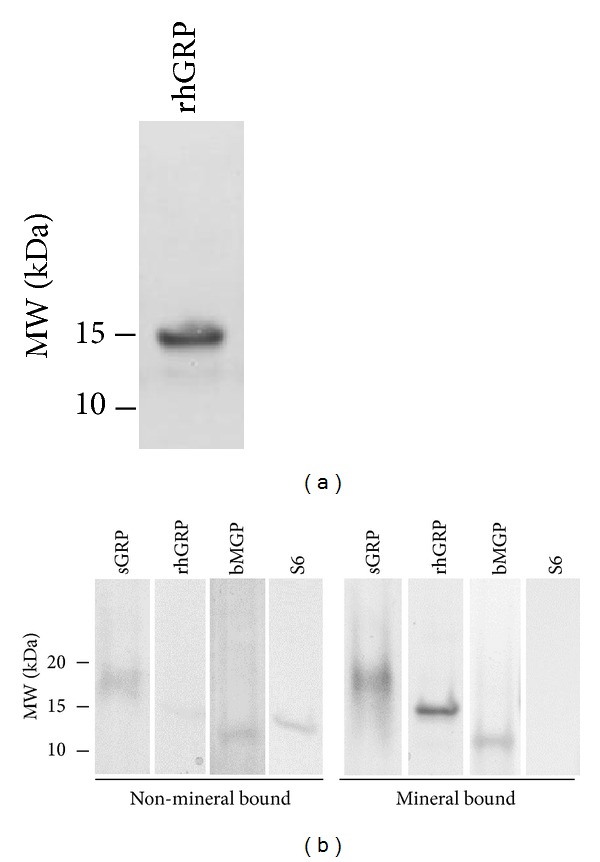
Both cGRP and ucGRP forms show* in vitro* mineral-binding affinity. (a) SDS-PAGE analysis of purified noncarboxylated recombinant human GRP-F1 (rhGRP) protein expressed in* E. coli*, detected with CBB. (b) SDS-PAGE analysis of non-mineral bound and mineral-bound proteins obtained from* in vitro* basic calcium-phosphate (BCP) protein mineral-binding assays, performed with purified rhGRP, sturgeon GRP (sGRP), bovine MGP (bMGP), and S6 ribosomal protein (S6). Relevant molecular weight markers (MW, kDa) are indicated on the left side of panels (a) and (b).

Human recombinant GRP-F1 protein (rhGRP) was expressed as a noncarboxylated secretory 107-aa protein, comprising the 74-aa GRP-F1 protein, with 33 aa of His_6_, epitope V (V5), and the TEV recognition site (TEV_RS) at its* N-terminus*. Purified rhGRP with an apparent molecular weight of 14 kDa on SDS-PAGE (Figure 9(a)) was further identified through LC-MS/MS analysis (Mass Spectroscopy facilities, ITQB-Lisbon, results not shown).

Protein fractions obtained in the PMC assays using noncarboxylated rhGRP, carboxylated sturgeon GRP (sGRP), S6, and bovine MGP (bMGP) were analyzed by SDS-PAGE. Results demonstrate that most of rhGRP, sGRP, and bMGP (used as positive control) were present in the demineralized fraction corresponding to the mineral-bound proteins, while S6, used as negative control, was predominantly found in the supernatant containing the non-mineral bound proteins (Figure 9(b)). These results confirm the BCP-binding capacity of both carboxylated and noncarboxylated GRP protein forms.

## 4. Discussion

*γ*-carboxylation of VKDPs is widely accepted to be determinant for their proper function, highlighting the importance of investigating *γ*-carboxylation status of GRP in humans. Human GRP carboxylation has been hypothesized on the basis of its high sequence similarity with sturgeon protein, previously shown to be *γ*-carboxylated, and the identification of specific domains and motifs conserved in other VKDPs [1]. In this work, we first investigated GRP *γ*-carboxylation status in human healthy tissues and further determined its association with ectopic calcification in cancers. Recently we have found additional GRP alternatively splice transcripts in human tissues (GRP-F5 and -F6, [9]) which were different from those previously described for mouse [7] and zebrafish [5, 8]. However, the GRP-F1 transcript was clearly shown to be the predominantly expressed variant in the control and cancer tissues analyzed. The newly developed conformation-specific antibodies were designed to detect the complete form of human secreted GRP (i.e., the GRP-F1 isoform), containing 15 Glu residues potentially *γ*-carboxylated. Since both GRP-F5 and -F6 contain exons 4 and 5 it would be possible that in certain conditions both cGRP pAb and ucGRP mAb colocalize different GRP isoforms. However, in this study the expression of GRP-F5 and -F6 was undetectable in the majority of the samples analyzed and their contribution for the GRP accumulation pattern was considered to be negligible. By using the new conformation-specific antibodies we were able to demonstrate the differential accumulation patterns of cGRP and ucGRP species in healthy skin and mammary gland tissues, their relation with neoplasias, and particular association with microcalcifications in skin and breast cancers. In healthy tissues, cGRP and ucGRP were found to be colocalized, suggesting an incomplete GRP *γ*-carboxylation status under normal physiological conditions. This result is consistent with the knowledge that all extra hepatic Gla proteins presently investigated are undercarboxylated in non-vitamin-K supplemented healthy individuals [13, 14]. Moreover, in tumor cells (both in BCC and IDC) cGRP was clearly lower than in non-affected areas, whereas ucGRP preferentially associated with tumor cells; high amounts of ucGRP were also found at sites of microcalcifications. Since conformation-specific GRP antibodies were produced against synthetic peptides covering small regions of GRP and possible *γ*-carboxylated Glu residues are present throughout the entire mature protein, the possibility of simultaneous detection of c/ucGRP protein forms cannot be completely discarded yet. Further characterization of monospecificity against native GRP species is currently under investigation. Nevertheless, these antibodies were found to have high specificity towards the respective synthetic GRP-related peptides used as antigens and clearly demonstrate different patterns of cGRP and ucGRP protein accumulation in the human tissues analyzed.

We have previously suggested that GRP may be a physiological inhibitor of soft tissue calcification accumulating at sites of mineral deposition [2], and the clear association of GRP with microcalcifications present in BCC and IDC further supports a global association of GRP with ectopic calcifications, independent of disease etiology. The presence of high amounts of ucGRP at sites of calcification, together with (i) the knowledge that Gla residues increase calcium binding capacity of VKDPs and (ii) that calcification inhibitors are known to accumulate at sites of mineral deposition [34–36], suggests a pivotal role for GRP in the regulation of mineralization that can be compromised in situations of low *γ*-carboxylase activity (e.g., by poor vitamin K status). In analogy, impaired carboxylation of MGP, leading to the accumulation of substantial amounts of ucMGP at sites of calcification, was previously suggested to be associated with a suboptimal capacity of arterial and skin calcification inhibition [18, 19, 36]. In concordance, our results show higher levels of GRP-F1 expression in IDC cases where ectopic calcifications were present. Although increasing sample sets, not yet available in our laboratory, would be required to clearly establish a relation between GRP-F1 expression levels and cancer development, it is interesting to note that expression pattern of GGCX, VKOR, and MGP was found highly similar to that of GRP-F1. This suggests that genes required for *γ*-carboxylation respond in a concerted manner according to demands of substrate and should not be limiting factors for carboxylation of VKDPs, such as GRP and MGP. However, increased gene expression might not reflect protein functionality, and impaired GGCX activity has been associated with insufficient *γ*-carboxylation of prothrombin in cancers [37, 38], while levels of GGCX mRNA have been shown either increased or heterogeneous among hepatocellular carcinomas [39]. Although prolonged subclinical vitamin K deficiency has been demonstrated as a risk factor for cancer development [40], a relation between vitamin K status or intake and decreased carboxylation of VKDPs is still controversial [24, 39]. However, the increased ucGRP accumulation in BCC and IDC and concomitant decreased of cGRP in relation to healthy tissues could be explained by decreased levels of vitamin K in tumor areas in contrast to non-tumorous, as previously reported [39]. Although a number of studies have shown that different forms of vitamin K (notably vitamin K2) exert antitumor activity on various rodent- and human-derived neoplastic cell lines [10, 30, 41], most of these effects have been correlated with increased *γ*-carboxylation of prothrombin leading to decreased levels of DCP [38, 39, 42]. Moreover, although levels of MGP mRNA have been suggested as a molecular marker for breast cancer prognosis, with overexpression and downregulation of MGP gene reported in different types of cancer and cell lines [reviewed in [25, 26]], its *γ*-carboxylation status in neoplasias remains unknown. Special attention should be given to the suggested therapeutic effect of vitamin K on cancer progression and to the potential detrimental effects of vitamin K antagonists [43] widely used in therapy of patients with cancer, on the functionality of VKDPs present in tumor tissues such as GRP and MGP.

Our protein-mineral complex* in vitro* studies provide insights into a possible mechanism explaining the accumulation of GRP at sites of pathological calcifications, since we demonstrated that both cGRP and ucGRP have calcium mineral-binding capacity and can directly bind BCP crystals. Similarly, MGP was shown to directly interact with HA crystals involving both phosphoserine and Gla residues; also for MGP the direct protein-HA interaction was suggested to be the mechanism underlying MGP arterial calcification inhibition [44]. Interactions between proteins and biological calcium crystals are believed to play a central role in preventing or limiting mineral formation in soft tissues and biological fluids, being determinant in several physiological processes and associated with pathological conditions. Additional studies are required to further clarify the role of cGRP and ucGRP species in calcium crystal nucleation and growth and to determine their precise mechanism of action at the molecular level.

Although further efforts will be made to highlight the relevance of GRP in cancer processes, we showed that GRP is associated with pathological mineralization in cancer and has the* in vitro* capacity to directly interact with calcium crystals. Our results emphasize that the involvement of this protein should be considered whenever conditions for correct carboxylation of VKDPs are affected. Furthermore, the measurement of carboxylation degree of Gla proteins, such as MGP, osteocalcin, and prothrombin, has been proposed as a marker for certain pathological conditions and vitamin K status [14, 40, 45, 46]. Further investigation aiming to correlate circulating levels and *γ*-carboxylation status of GRP with the degree of calcification and disease progression are currently in progress in our labs. We expect that our work will contribute to the evaluation of GRP potential usage as an additional marker for ectopic calcification.

## 5. Conclusions

Here we report the *γ*-carboxylation status of GRP-F1 in human healthy tissues and its association with skin and breast cancers. The new conformation-specific GRP antibodies enable us to demonstrate that in healthy tissues, cGRP and ucGRP were found to be colocalized, suggesting an incomplete GRP *γ*-carboxylation status under normal physiological conditions, while ucGRP was the predominant form associated with tumor cells.

Our results strengthen the previously reported association of GRP with ectopic calcifications, which are particularly relevant in the diagnosis of breast tumors. Our findings suggest that GRP may represent a new target for the anticancer potential of vitamin K, while the degree of GRP *γ*-carboxylation might be useful as a potential marker for vitamin K status and ectopic calcification occurrence.

## Figures and Tables

**Table 1:** Histopathological features of skin cancer samples.

Sample ID	Cancer type	Histological grade	Localization	Calcification	Age	Gender	Purpose
SC1	SC^∗1^	Well differentiated, keratinizing	Lower limb	Absent	86	F	Gene expression
SC2	BCC^∗2^	Nodular	Trunk	Absent	82	F	Gene expression/IHC
SC3	BCC	Nodular, pigmented	Trunk	Absent	67	M	Gene expression/IHC
SC4	BCC	Nodular, pigmented, and cystic	Trunk	Absent	81	F	Gene expression
SC5	BCC	Superficial, cystic	Trunk	Absent	54	F	Gene expression
SC6	BCC	Nodular, cystic	Face	Absent	85	F	IHC
SC7	BCC	Nodular	Scalp	Absent	39	F	IHC
SC8	BCC	Nodular	Upper limb	Absent	97	F	IHC
SC9	BCC	Nodular	Auricular pavilion	Absent	82	F	IHC
SC10	BCC	Nodular, pigmented	Face	Light	70	F	IHC
SC11	BCC	Nodular	Lower limb	Moderate	61	M	IHC

^∗1^Spinocellular carcinoma; ^∗2^basal cell carcinoma.

**Table 2:** Histopathological features of breast cancer samples.

Sample ID	Cancer type	Histological grade	Calcification	Age	Purpose
BC1	IDC^∗1^-Mucinous	Moderately differentiated	Light	64	Gene expression
BC2	IDC	Moderately differentiated	Light	66	Gene expression
BC3	IDC	Moderately differentiated	Absent	82	Gene expression
BC4	IDC	Well differentiated	Absent	89	Gene expression
BC5	IDC	Well differentiated	Light	53	IHC
BC6	IDC	Moderately differentiated	Moderate	52	IHC
BC7	IDC	Well differentiated	Massive	54	IHC
BC8	IDC	Poorly differentiated	Absent	76	IHC
BC9	IDC	Moderately differentiated	Absent	49	IHC
BC10	IDC	Moderately differentiated	Absent	47	IHC
BC11	IDC	Well differentiated	Massive	55	IHC

^∗1^IDC: invasive ductal carcinoma.

**Table 3:** Primers for PCR amplifications.

Primer name	Sequence (5′ to 3′)	Purpose/amplicon
dT-adapter	ACGCGTCGACCTCGAGATCGATG(T)_13_	RT

A	GTCCCCCAAGTCCCGAGATGAGG	qPCR/GRP-F1
B	CCTCCACGAAGTTCTCAAATTCATTCC	

C	TCCTGGACGGAGCCCCTA	qPCR/GRP-F5, F6

D	GCTTCTGCCTGTTTTCCACTTCAC	qPCR/GRP-F5

E	GCTTCTGCCTGTTTTCCATAGACA	qPCR/GRP-F6

MGP_F	TGGAGGCTGGCACCTGATTTTG	qPCR/MGP
MGP_R	AAAAGGGGTGCAGCCAGACAAG	

GGCX_F	TTACACAGAGTCGGCGATGGAAGGAT	qPCR/GGCX
GGCX_R	AGTGTGGTTGTCTAGGCTGCTCTTGAT	

VKORC1_F	AGGGCAAGGCTAAGAGGCACTGAG	qPCR/VKORC1
VKORC1_R	CTGGGCAATGGAAAGAGCTTTGGAGAC	

OPN_F	ACGGACCTGCCAGCAACCGAAGT	qPCR/OPN
OPN_R	TACTGGATGTCAGGTCTGCGAAA	

TNF*α*_F	AGGGCCTGTACCTCATCTACTCCCA	qPCR/TNF*α*
TNF*α*_R	AGCTGGAAGACCCCTCCCAGATAGA	

18S_F	GGAGTATGGTTGCAAAGCTGA	qPCR/18S
18S_R	GGAGTATGGTTGCAAAGCTGA	

GAPDH_F	AAGGTGAAGGTCGGAGTCAACGGA	qPCR/GAPDH
GAPDH_R	TCGCTCCTGGAAGATGGTGATGGG	

HsGRPCDS1_Fw	GACGCCTGGTCTGCCTTGTGGG	cDNA cloning

HsGRPCDS3_Rv	CTTGGGAACGAAGCCAGGGGA	cDNA cloning

HsGRPCDS2_Fw	TCCTGGACGGAGCCCCTACCT	cDNA cloning

HsGRPEx5R2	ACACGGGGATGCCAATGGTGCTAC	cDNA cloning

ReHsGRP_CFw	*CACCTCCCCCAAGTCCCGAGATGA	pET151/D-TOPO cloning

ReHsGRP_Rv	TCACGTGTGGTGGCGGTTGTAGA	pET151/D-TOPO cloning

*Overhang sequence to pET151/D-TOPO for directional cloning.
